# Protein Arginine Methyltransferase 5 Functions *via* Interacting Proteins

**DOI:** 10.3389/fcell.2021.725301

**Published:** 2021-08-27

**Authors:** Zhenzhen Liang, Chaowei Wen, Heya Jiang, Shumei Ma, Xiaodong Liu

**Affiliations:** ^1^School of Public Health and Management, Wenzhou Medical University, Wenzhou, China; ^2^NHC Key Lab of Radiobiology, Jilin University, Changchun, China; ^3^Key Laboratory of Watershed Science and Health of Zhejiang Province, Wenzhou Medical University, Wenzhou, China

**Keywords:** interacting proteins, methylation, post-translational modification, cell cycle, cell death, PRMT5

## Abstract

The protein arginine methyltransferases (PRMTs) are involved in such biological processes as transcription regulation, DNA repair, RNA splicing, and signal transduction, etc. In this study, we mainly focused on PRMT5, a member of the type II PRMTs, which functions mainly alongside other interacting proteins. PRMT5 has been shown to be overexpressed in a wide variety of cancers and other diseases, and is involved in the regulation of Epstein-Barr virus infection, viral carcinogenesis, spliceosome, hepatitis B, cell cycles, and various signaling pathways. We analyzed the regulatory roles of PRMT5 and interacting proteins in various biological processes above-mentioned, to elucidate for the first time the interaction between PRMT5 and its interacting proteins. This systemic analysis will enrich the biological theory and contribute to the development of novel therapies.

## Introduction

Protein post-translational modification (PTM) is critical for proteome diversification, which contributes to the regulation of protein function and cell signal transduction. Among PTM, the protein methylation, modified by protein lysine methyltransferases (PKMTs) and protein arginine methyltransferases (PRMTs) targeting at lysine and arginine residues, respectively, has attracted attention, (Dilworth and Barsyte-Lovejoy, 2019), however, protein arginine methylation has not been fully studied.

In eukaryotes, monomethylation, and dimethylation modification of arginine, are correspondent to the formation of monomethylated arginine, symmetrical dimethylarginine and asymmetric dimethylarginine (Guccione and Richard, 2019). At present, nine kinds of human-derived PRMTs have been discovered and divided into three types according to the catalytic activity: type I PRMTs are responsible for the catalytic generation of monomethylated arginine and asymmetric dimethylarginine, including PRMT1, PRMT2, PRMT3, PRMT4, PRMT6, and PRMT8; type II PRMTs are responsible for the generation of monomethylated arginine and symmetric dimethylarginine, including PRMT5 and PRMT9; PRMT7 is the sole type III PRMT enzyme, which is only responsible for monomethylation of arginine (Jain and Clarke, 2019). PRMTs can catalyze the methylation of different types of proteins, such as histones H2AR3, H3R4, H3R8, H4R3, H3R8, H3R26, H4R17, etc.; DNA repair factor TP53BP1, RNA binding protein SREBP1; transcription factors FOXO1, SOX2, PAX7; RNA splicing factor SF3B2 and SAP145, etc., (Kuenne et al., 2015; Yang et al., 2015; Hadjikyriacou and Clarke, 2017; Muhammad et al., 2017; Huang L. et al., 2018).

Methylation either changes the structure of chromatin and leads to chromatin remodeling, or provides binding sites for transcriptional regulators (Pahlich et al., 2006). The methylation of arginine residues causes the replacement of the hydrogen on the amino, which affects the formation of hydrogen bonds. This does not change the charge of the proteins, but increases the hydrophobicity and changes the huge arginine side chain, having impacts on the interaction between proteins (Zhu and Rui, 2019).

### The General Function of PRMT5

Among the nine members of the PRMT family, PRMT5, PRMT1, and CARM1 are most highly expressed in cancer (Kim and Ronai, 2020). PRMT5 is the main type II PRMTs, and studies have confirmed that the abnormal expression of PRMT5 is closely related to the occurrence and development of various diseases. PRMT5 is overexpressed in solid tumors and hematological tumors, and related to poor prognosis (Gullà et al., 2018; Jarrold and Davies, 2019; Tan et al., 2020). As a protein arginine methyltransferase, PRMT5 catalyzes the methylation of targeted proteins, and either inhibits the transcription of tumor suppressors (such as p53, E2F-1, ST7, NM23, and CASP4) to promote tumor occurrence, (Pal et al., 2004; Zhu and Rui, 2019) or is involved in the activation of transcription factors. Cytoplasmic PRMT5 induces arginine methylation of various transcription factors (such as NF-κB/p65, PDCD4, SREBP1, KLF4, etc.) and these transcription factors translocate into the nucleus and regulate target genes. Nuclear PRMT5 is also directly recruited to promoter regions of target genes to enhance cellular proliferation and oncogenesis (Shailesh et al., 2018). PRMT5 has become an essential epigenetic regulator for cell proliferation and tumor development.

### The Interacting Proteins of PRMT5

The interacting proteins of PRMT5 were screened based on the public databases STRING^1^, IntAct^2^, MINT^3^, HPRD^4^, PINA^5^, and BioGRID^6^. STRING covers the largest number of organisms and uses the widest breadth of input sources, including automated text-mining and computational predictions (Szklarczyk et al., 2017). In IntAct database, all protein interaction information comes from literature reports or users’ direct online submission. MINT mainly stores mammals’ protein-protein interacting (PPI) data extracted from scientific literature and annotated in the database by professional curators (Calderone et al., 2020). HPRD database only contains human PPI and is the largest human PPI database from literature mining containing PTM, subcellular localization, domain, and other information. PINA database is the integration of PPI data in MINT and HPRD databases. BioGRID is mainly used to store protein and genetic interaction data of human and other species reported in biomedical literature (Oughtred et al., 2019).

We searched the interacting proteins of PRMT5 in these databases, limiting the species to human. The results are listed in Supplementary Table 1. Based on retrieved interacting proteins, we drew an upset graph to display the number of proteins in each database. As shown in Supplementary Figure 1, two proteins were found to interact with PRMT5 in all of the six databases, namely WDR77 and CLNS1A. In fact, WDR77 (also called MEP50) and CLNS1A are non-catalytic components of methyl complex, and they form methyl complex together with PRMT5 and function as co-activators for PRMT5 activity. Recent studies have shown that the activity of PRMT5 alone was about 50% of PRMT5 in complex with WDR77 (Yi et al., 2020).

### Enrichment Analysis on PRMT5 Interacting Proteins (PIPs)

**FIGURE 1:**
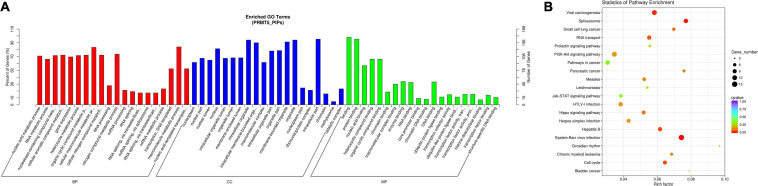
Functional GO and KEGG pathway enrichment analysis of PRMT5 interacting proteins. **(A)** Plots of significantly enriched GO terms of PRMT5 interacting proteins for biological processes (BP), cellular components (CC) and molecular function (MF). **(B)** Significantly enriched KEGG pathway terms of PRMT5 interacting proteins.

Based on the above PIPs, we found 155 proteins that were listed in at least two databases. Then, we performed a functional enrichment analysis on those 155 PIPs (Figures 1A,B Detailed results are shown in Supplementary Tables 2, 3). We found: (1) the molecular functions of PIPs included protein binding, nucleic acid binding, chromatin binding, transcription cofactor activity, RNA binding, enzyme binding, etc.; (2) for biological process, PIPs were involved in nucleic acid metabolism, RNA metabolism, gene expression, cellular aromatic compound metabolism, RNA splicing, etc.; (3) in terms of cell components, PIPs were mainly involved in the formation of nucleoplasm, nuclear lumen, organelle lumen, methylosome, etc. KEGG pathway analysis results revealed that the top five most enriched pathways for PIPs were Epstein-Barr virus infection, spliceosome, viral carcinogenesis, hepatitis B and cell cycle.

### The Biological Functions of PRMT5 Together With Interacting Proteins

In view of the above-mentioned signaling pathways, we further analyzed the molecular mechanisms of PRMT5 and PIPs by virtue of interacting proteins.

#### Epstein-Barr Virus Infection and Viral Carcinogenesis

The Epstein-Barr virus (EBV) is characterized by specifically infecting humans and some primate B cells *in vivo* and *in vitro*. EBV is an oncogenic virus that is associated with a broad spectrum of human malignancies. The combination of PRMT5 and EBV protein EBNA1 is important for the replication and mitotic separation of the viral genome. EBNA1 persists in the viral genome in the infected circulating B lymphocytes, which may lead to malignant transformation (Shire et al., 2006). In addition to EBNA1, EBNA2 is also a substrate of PRMT5. EBNA2 features an Arg-Gly repeat (RG) domain which is a known target for PRMT5. Study has found that EBNA2 and PRMT5 are co-localized in the nucleus. PRMT5 binds to the RG domain of EBNA2 and catalyzes the symmetrical dimethylation of arginine residues. This biochemical event increases the occupancy of EBNA2 in the target promoter and leads to the enhancement of transcriptional activity, indicating PRMT5 plays a crucial role in enhancing EBNA2-mediated transcription (Liu et al., 2013).

Studies also found that EBV utilizes PRMT5 to target and silence genes with important tumor suppressor properties during B-cell immortalization. PRMT5 overexpression may be an important mechanism to promote the immortalization of B cells after EBV infection (Alinari et al., 2015). In addition, compared with cells before infection, a three to fourfold increase in rRNA synthesis was detected in EBV immortalized B cells, and further research found that PRMT5 regulated rRNA promoter activity in EBV-transformed B cells (Majumder et al., 2010).

#### Hepatitis and Hepatocellular Carcinoma

Inflammation might contribute to the development and progression of malignancies (Diakos et al., 2014). PRMT5 plays roles in virus activation. ORF59, an early protein of viral reactivation, binds to the N-terminal domain of PRMT5, and competitively destroys the binding of PRMT5 and COPR5, leading to the decrease of H4R3 symmetric dimethylarginine (SDMA) level and the increase of viral gene transcription (Strahan et al., 2017). PRMT5 inhibits the expression of viral antigen and cccDNA by increasing the SDMA of cccDNA-bound H4R3. Actually, PRMT5 not only inhibits HBV DNA transcription, but also inhibits the production of HBV core particle DNA in a methyltransferase activity-independent manner. PRMT5 blocks polymerase by binding to the reverse transcriptase ribonuclease H region of the polymerase, interfering with the capsizing of genomic RNA (Zhang et al., 2017). In addition, HBV core protein (HBc) interacts with PRMT5 and MEP50 through its C-terminal arginine-rich domain, and arginine methylation could regulate the shuttle of HBC protein from nucleus to cytoplasm. Downregulation of PRMT5 and MEP50 decreased the level of HBC protein in the nucleus. Inhibition of SDMA could prevent the nuclear input of HBC protein (Lubyova et al., 2017).

**FIGURE 2:**
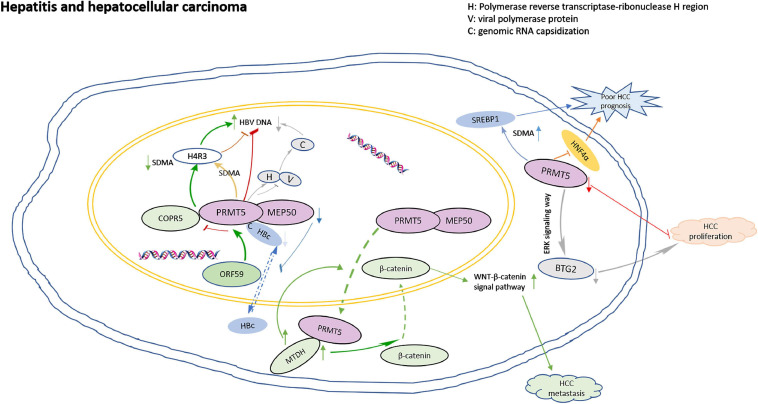
The model for PRMT5 roles in regulation of hepatitis and hepatocellular carcinoma. ORF59 binds to the N-terminal domain of PRMT5, competitively destroying the binding of PRMT5 and COPR5, leading to the decrease of H4R3 SDMA levels and the increase of viral gene transcription; PRMT5 blocks polymerase by binding to the reverse transcriptase ribonuclease H region of the polymerase, interfering with the capsizing of genomic RNA; HBV core protein (HBc) interacts with PRMT5 and MEP50 through its C-terminal arginine-rich domain, and arginine methylation could regulate the nuclear to cytoplasmic shuttle of HBC protein. Down-regulation of PRMT5 and MEP50 decreased the level of HBC protein in nucleus. When MTDH is overexpressed in HCC cells, PRMT5 transfers from the nucleus to the cytoplasm, and then β-catenin transfers from the cytoplasm to the nucleus, and the WNT-β-catenin signaling pathway is upregulated. MTDH-PRMT5 complex could promote the metastasis of HCC by regulating WNT-β-catenin signaling pathway. PRMT5 leads to the down regulation of tumor suppressor BTG2 expression through ERK pathway, and consequently accelerated HCC proliferation. PRMT5 antagonizes the expression of HNF4α and resulted in poor prognosis.

PRMT5 is overexpressed in hepatocellular carcinoma (HCC), and associated with aggressive clinicopathological parameters, such as poor differentiation, higher incidence of hepatic vein invasion, larger tumor size, higher AFP levels and worse prognosis (Shimizu et al., 2017; Jeon et al., 2018). PRMT5 interacts with MTDH in HCC cells. When MTDH is overexpressed in HCC cells, PRMT5 transfers from the nucleus to the cytoplasm, then β-catenin transfers from the cytoplasm to the nucleus, and the WNT-β-catenin signaling pathway is up-regulated. MTDH-PRMT5 complex could promote the metastasis of HCC by regulating WNT-β-catenin signaling pathway (Zhu et al., 2020). PRMT5 leads to the down-regulation of tumor suppressor BTG2 expression through ERK pathway, and consequently accelerated HCC proliferation (Jiang et al., 2018). PRMT5 also regulates the cancer growth through methylation of SREBP1 and tissue mSREBP1a R321 SDMA was associated with poor prognosis of HCC (Liu et al., 2016). In addition, HNF4α has been shown to re-differentiate HCC cells into hepatocytes and inhibits EMT, thereby blocking the occurrence of liver cancer, however, PRMT5 antagonizes the expression of HNF4α and helps maintain liver cancer stem cells (Wright et al., 2021). The model for PRMT5 roles in regulation of hepatitis and HCC is shown in Figure 2.

#### Spliceosome

In humans, more than 95% of multi-exon precursor mRNA transcripts undergo alternative splicing. As a splicing modulator, PRMT5 mediates SDMA of the spliceosome Sm protein (Ratovitski et al., 2015). The Sm proteins belong to conserved protein family with Sm motifs. The Sm core proteins, including SmB, SmB′, SmN, SmD1, SmD2, SmD3, SmE, SmF, and SmG, stably bind with small nuclear RNAs (snRNAs) and other proteins to form small nuclear ribonucleoproteins (snRNPs) in the nucleus (Li X. et al., 2020). In higher eukaryotes, PRMT5 catalyzes the symmetric dimethylation of Arg residue in key components of the spliceosome.

Study shows that pICln recruits Sm proteins into the PRMT5 complex for methylation, then the methylated Sm proteins are loaded onto snRNAs to form snRNPs (Guderian et al., 2011). snRNP assembly is thought to occur in two stages. The early stage is dominated by the PRMT5 complex, and the newly translated Sm D2 protein is thought to be still attached to the ribosome. The formation of Sm D2/D1 dimers and their binding to pICln ensure their release and subsequent delivery to the PRMT5 complex (Paknia et al., 2016). The loss of the Sm protein from the SDMA prevents the recruitment of the NineTeen complex and the initiation of spliceosome activation (Deng et al., 2016). In addition, as a component of 20S methylosome, PRMT5 not only methylates SmD1 and SmD3, but also induces their interaction with SMN proteins, which is essential for the cytoplasmic assembly of snRNPs (Friesen et al., 2001; Meister et al., 2001).

**FIGURE 3:**
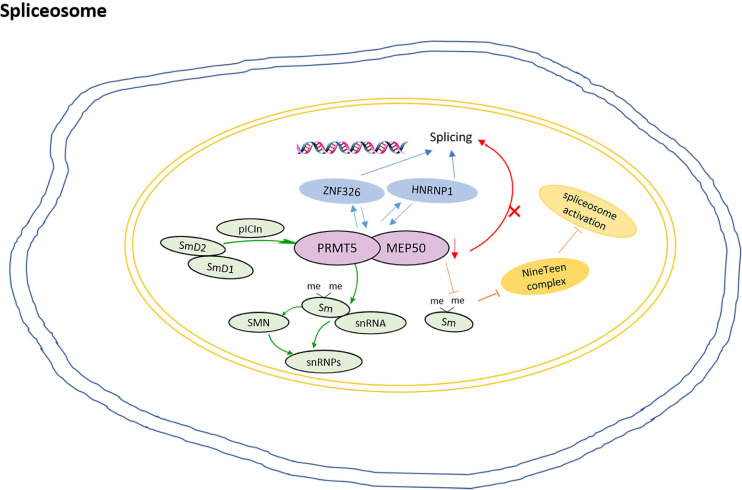
The model for PRMT5 roles in regulation of spliceosome. pICln recruits Sm proteins into the PRMT5 complex for methylation, then the methylated Sm proteins are loaded onto snRNAs to form snRNPs; The formation of Sm D2/D1 dimers and their binding to pICln ensures their release and subsequent delivery to the PRMT5 complex; The loss of Sm protein SDMA prevents the recruitment of the NineTeen complex and the initiation of spliceosome activation; ZNF326 interacts with PRMT5 and WDR77, and HNRNPH1, the loss of PRMT5 and WDR77 leads to the overall defect of alternative splicing.

In the human breast cancer MDA-MB-231 cell, PRMT5 and WDR77 show high expression and nuclear localization. ZNF326 is a zinc finger protein that moves with the core mRNP particles during transcription and regulates splicing by controlling the elongation rate of transcription. ZNF326 interacts with PRMT5 and WDR77; HNRNPH1, a component of mRNP particles, also interacts with PRMT5/WDR77 complexes. The loss of PRMT5 and WDR77 leads to the overall defect of alternative splicing, which is mainly due to the change of exon inclusion or exclusion through exon skipping (SE), (Rengasamy et al., 2017) and this change may be caused by the loss of methylation of splice proteins (Bezzi et al., 2013). Moreover, PRMT5 interacts with TDRD6 in spermatocytes at prophase of meiosis. TDRD6 acts as a tissue-specific mediator of PRMT5 for SmB methylation in testis and TDRD6-deficient primary spermatocytes exhibit extensive splicing disturbance (Akpınar and Lesche, 2017). The model for roles of PRMT5 in the regulation of spliceosome is shown in Figure 3.

#### Cell Cycle and Apoptosis

PRMT5 regulates cell cycle and apoptosis by the following aspects.

Firstly, PRMT5 regulates cell cycle and apoptosis by methylating histones. In acute lymphoblastic leukemia (ALL), PRMT5 promotes the proliferation of lymphocytes by inhibiting apoptosis, and the down-regulation of H4R3sme2 by PRMT5 silencing induced ALL differentiation from the pre-B to immature B stage (Mei and Zhang, 2019). In addition, the inhibition of PRMT5-mediated H3K27 methylation contributes to cell cycle progression (Liu F. et al., 2020).

Secondly, PRMT5 functions in a DNA damage responsive co-activator complex that interacts with p53, and is responsible for the methylation of p53. PRMT5 depletion caused an increase of sub-G1 cells during the DNA damage response, simultaneously, compared with wild-type p53, a p53 mutant compromised in arginine methylation may affect the cell cycle (Radzisheuskaya et al., 2019). In addition, the role of PRMT5 in the process of DNA damage repair is also mediated by 53BP1, and PRMT5 activity may be the main determinant of whether cells undergo DNA repair or apoptosis in response to DNA damage (Jansson et al., 2008). Moreover, reducing PRMT5 activity up-regulates exon skipping and intron retention events, which has an influence on the genes involved in DNA repair pathway. Therefore, the loss of PRMT5 activity leads to endogenous DNA damage which triggers p53 activation and induces apoptosis (Hwang et al., 2020). PRMT5 also inhibits cyclin D1T286A-induced apoptosis in a MEP50 phosphorylation-dependent manner, and cyclin D1T286A/CDK4 promotes p53-dependent PRMT5 methylation (Tan et al., 2019).

Thirdly, PRMT5 promotes cell cycle progression by up-regulating cell cycle proteins such as β-catenin, Cyclin D1, CDK4/6, and CCND1/D2/E1 (Li et al., 2015). PRMT5 inhibition resulted in a decrease in the proportion of G1 and S phases, an increase in the proportion of the G2/M phase, and an increase in apoptosis of sub-G1 (Wei et al., 2012). Knockdown of PRMT5 causes an increase of G1 phase of HCC cells, and a corresponding decrease in S and G2/M phases (Fong et al., 2019).

Fourthly, PRMT5 regulates cell cycle and apoptosis by directly or indirectly affecting PIPs. Both PRMT5 and PRMT1 interact with CFLAR_L_ which is a key anti-apoptotic protein. PRMT5 and PRMT1 regulate the polyubiquitination and degradation of CFLAR_L_ by influencing the interaction between CFLAR_L_ and ITCH, then regulate the apoptosis of non-small cell lung cancer cells by regulating CFLAR_L_ (Zhang et al., 2015). PRMT5 and Myc protein have obvious co-localization pattern, mainly in the nucleus. Inhibition of the specific interaction between PRMT5 and Myc prevents the growth of medulloblastoma cells and promotes the apoptosis of Myc-dependent tumors (Li et al., 2019). The depletion of PRMT5 impacts the binding of SRSF1 to mRNAs and proteins, leads to alternative splicing of multiple essential genes and, consequently, death of human acute myeloid leukemia cells (Ichikawa et al., 2020). In addition, PRMT5 interacts with Zili and Vasa, and directly catalyzes the SDMA of Vasa and Zili. The deletion of PRMT5 leads to the dysregulation of the expression of Vasa and Zili, and triggers germ cell apoptosis (Zhu J. et al., 2019).

Fifthly, PRMT5 regulates cell cycle and apoptosis through multiple signaling pathways. When PRMT5 is inhibited, the down-regulation of Akt and PTEN will induce apoptosis (Chaturvedi et al., 2019). In diffuse large B-cell lymphoma (DLBCL), the expression of PRMT5 is increased by B-cell receptor (BCR) signaling. BTK-NF-κB signaling induces PRMT5 transcription in activated DLBCL cells while BCR downstream PI3K-AKT-MYC signaling up-regulates PRMT5 expression in DLBCL cells. PRMT5 activates PI3K-AKT signaling and promotes cell cycle progression, suggesting a feedback regulatory mechanism to enhance cell survival and proliferation (Zhu F. et al., 2019). In the meantime, PRMT5 directly interacts with Akt and promotes cell proliferation by regulating Akt activity instead of PTEN and mTOR signaling pathways. Targeting PRMT5/Akt signaling axis prevents cell proliferation (Zhang et al., 2019). When PRMT5 is inhibited, the phosphorylation of Akt/GSK3β and the expression of downstream target cyclin E1 and cyclin D1 are significantly reduced, indicating that blocking PRMT5 activity can prevent cell proliferation and cell cycle progression (Li Y.H. et al., 2020). PRMT5 also enhances NF-κB activation by targeting key anti-apoptotic genes (such as BCLXL and c-IAP1), thereby inhibiting apoptosis and maintaining proliferation (Hu et al., 2018). At the same time, PRMT5 controls the expression of survival genes such as CYCLIN D1, c-MYC, and SURVIVIN by promoting WNT/β-CATENIN and AKT/GSK3β. PRMT5 promotes the survival of lymphoma cells, and its inhibitory effect induces lymphoma cell death (Chung and Karkhanis, 2019). In addition, PRMT5 can stimulate liver glucose metabolism, (Tsai et al., 2013) and activate CDK4-pRB-E2F-mediated transcription under glucose induction to promote HCC cell cycle progression (Yang et al., 2016).

**FIGURE 4:**
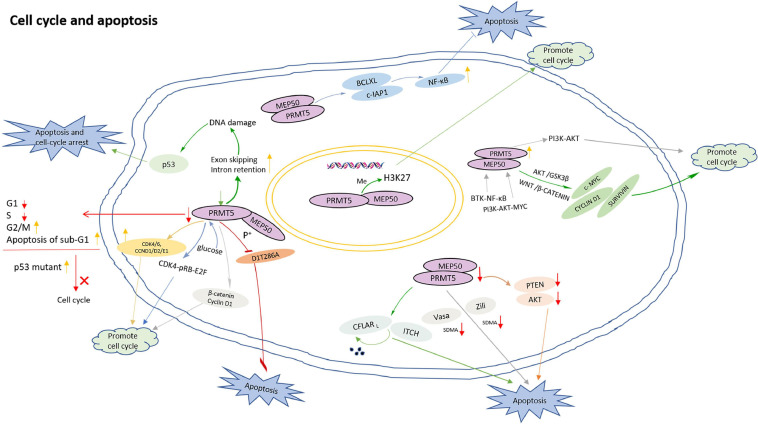
The model for PRMT5 roles in regulation of cell cycle and apoptosis. PRMT5-mediated H3K27 methylation inhibition contributes to cell cycle progression; PRMT5 depletion caused an increase of sub-G1 cells during the DNA damage response; Loss of PRMT5 activity leads to endogenous DNA damage which triggers p53 activation and induces cell apoptosis; Compared with wild-type p53, a p53 mutant compromised in arginine methylation has altered effects on the cell cycle; PRMT5 inhibits cyclin D1T286A-induced apoptosis in a MEP50 phosphorylation-dependent manner; PRMT5 promotes cell cycle progression by up-regulating β-catenin, Cyclin D1, CDK4/6, and CCND1/D2/E1; PRMT5 inhibition resulted in a decrease in the proportion of cells in G1 and S phases, an increase in the proportion of cells in the G2/M phase, and an increase in apoptosis of sub-G1; By influencing the interaction between CFLAR_*L*_ and ITCH, PRMT5 regulates the polyubiquitination and degradation of CFLAR_*L*_, regulating the apoptosis of non-small cell lung cancer cells; PRMT5 interacts with Zili and Vasa, and directly catalyzes the SDMA of Vasa and Zili. The deletion of PRMT5 leads to the dysregulation of the expression of Vasa and Zili, and lead to germ cell apoptosis; When PRMT5 is inhibited, the down regulation of various signal molecules such as Akt and PTEN will induce apoptosis; PRMT5 can stimulate liver glucose metabolism, and activate CDK4-pRB-E2F-mediated transcription under glucose induction to promote HCC cell cycle progression; PRMT5 activates PI3K-AKT signaling and promotes cell cycle progression.

The model for PRMT5 roles in regulation of cell cycle and apoptosis is shown in Figure 4.

#### Other Signaling Pathways

In addition to the above-mentioned main pathways, PRMT5 also participates in the regulation of the following signals:

Firstly, PRMT5 is involved in cell proliferation. TAp63, the phosphorylation target of GAK (Cyclin G-related kinase), regulates the transcription of LCE1C gene, PRMT5 interacts with LCE1C, and LCE1C promotes the cytoplasmic retention of PRMT5 or the transport of PRMT5 from nucleus to cytoplasm through direct interaction, thereby preventing the nuclear function of PRMT5 (Yabuta et al., 2018). It is worth noting that some studies have shown that the subcellular localization of PRMT5 may change its characteristics. In prostate cancer, the nuclear PRMT5 is responsible for proliferation inhibition and is related to the prolongation of survival rate while the cytoplasmic PRMT5 promotes cell proliferation (Lattouf et al., 2019). In addition, the direct binding of PRMT5 and c-Myc transcriptionally inhibits the expression of a series of genes, including PTEN, p63, CDKN2C, and so on, thus promoting the proliferation of gastric cancer cells (Liu M. et al., 2020).

Secondly, PRMT5 is involved in immune hematopoietic regulation. PRMT5 is essential for B cell development. It regulates the fidelity of B cell transcription and splicing, protects B cell from apoptosis and promotes proliferation (Litzler et al., 2019). The depletion of PRMT5 activates mTOR signaling and increases protein synthesis in hematopoietic stem cells (Hwang et al., 2020). Prmt5 is essential for the differentiation of lymphoid progenitor cells and T cells in zebrafish, and the methyltransferase activity of Prmt5 is necessary for its hematopoietic function (Quillien et al., 2021).

Thirdly, PRMT5 is involved in tumor angiogenesis, cell invasion and metastasis. PRMT5 interacts directly with HOXC10, promoting the enrichment of H3R2me1 and H3R2me2s on the VEGFA promoter, and then recruiting WDR5 for subsequent methylation of H3K4 on the VEGFA promoter, which eventually leads to the transcription of VEGFA and promotes angiogenesis in glioma (Tan et al., 2018). PRMT5 promotes the metastasis of laryngeal carcinoma by activating Wnt4/β – catenin signaling pathway (Wang et al., 2020). Inhibition of PRMT5 reduces the transcription of Twist 1 mediated by H3K4me3 and delays the metastasis of squamous cell carcinoma of head and neck (Fan et al., 2020). Moreover, macrophage-capping protein (CAPG) has been shown to promote cancer metastasis, STC-1 enhances the invasion of cancer cells by activating PI3K pathway. PRMT5 directly binds to CAPG, and CAPG regulate STC-1 transcription by competing with PRMT5 to enhance breast cancer metastasis (Huang S. et al., 2018). In addition, the knock-down of PRMT5 could inhibit tumor growth and lung metastasis by the up-regulation of LKB1 and p-AMPK, and the down-regulation of p-mTOR. PRMT5 may act as a tumor inducer in esophageal squamous cell carcinoma by regulating the LKB1/AMPK/mTOR signaling pathway (Chen et al., 2021). Epithelial-mesenchymal transition (EMT) is an important process triggered during cancer metastasis. PRMT5 is identified to be crucial for the regulation of EMT transcription factors (Lin and Wu, 2020). Overexpressed PRMT5 promotes EMT by activating EGFR/AKT/β-catenin signaling in pancreatic cancer cells (Ge et al., 2020), as well as lung cancer cells (Huang et al., 2021).

**FIGURE 5:**
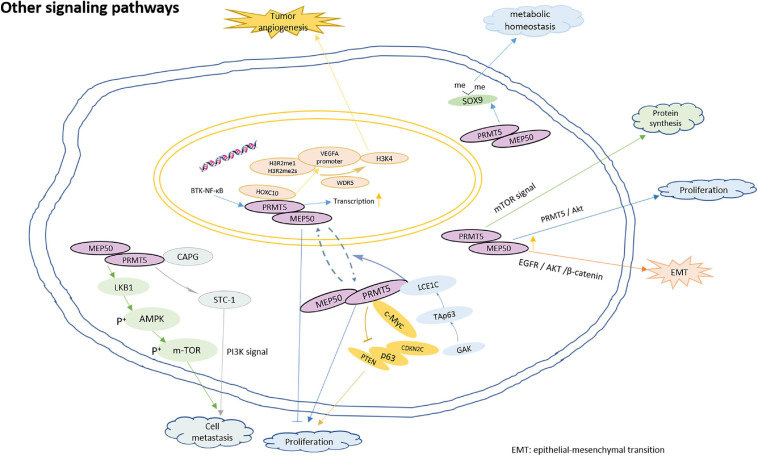
The model for PRMT5 roles in regulation of other biological functions. PRMT5 interacts with LCE1C, and LCE1C promotes the cytoplasmic retention of PRMT5 or the transport of PRMT5 from nucleus to cytoplasm through direct interaction, thereby preventing cell proliferation; The direct binding of PRMT5 and c-Myc transcriptionally inhibits the expression PTEN, p63, CDKN2C, and so on, thus promoting the proliferation of gastric cancer cells; The depletion of PRMT5 activates mTOR signaling and increase protein synthesis in hematopoietic stem cells; PRMT5 interacts directly with HOXC10, promoting the enrichment of H3R2me1 and H3R2me2s on the VEGFA promoter, and recruiting WDR5 for subsequent methylation of H3K4 on the VEGFA promoter, which eventually leads to the transcription of VEGFA and promotes angiogenesis in glioma; PRMT5 promotes the metastasis of laryngeal carcinoma by activating Wnt4/β – catenin signaling pathway; PRMT5 directly binds to CAPG, and CAPG regulate STC-1 transcription by competing with PRMT5 to enhance breast cancer metastasis; Knocking down PRMT5 could inhibit tumor growth and lung metastasis by up-regulating the expression of LKB1 and p-AMPK, and down-regulating the expression of p-mTOR; overexpressed PRMT5 promotes EMT by activating EGFR/AKT/β-catenin signaling; PRMT5 could improve SOX9 stability by dimethylating the protein, which contributes to maintain the matrix metabolic homeostasis of the chondrocytes.

Fourthly, PRMT5 is involved in the regulation of homeostasis. Cells subjected to genotoxic stress need to reestablish reduction-oxidation (redox) homeostasis to scavenge genotoxic stress-induced reactive oxygen species (ROS). PRMT5 contributes epigenetically to JDP2/β-catenin-activated GSH metabolism upon genotoxic stress (Cao et al., 2019). In addition, PRMT5 could improve SOX9 stability by dimethylating the protein, which contributes to maintain the matrix metabolic homeostasis of the chondrocytes (Sun et al., 2019). Moreover, PRMT5 plays an important role in T cell homeostasis and activation-induced expansion. PRMT5 is necessary to maintain cytokine signaling for T cell survival and proliferation (Tanaka et al., 2020). The model for PRMT5 roles in regulation of multiple biological functions is shown in Figure 5.

**FIGURE 6:**
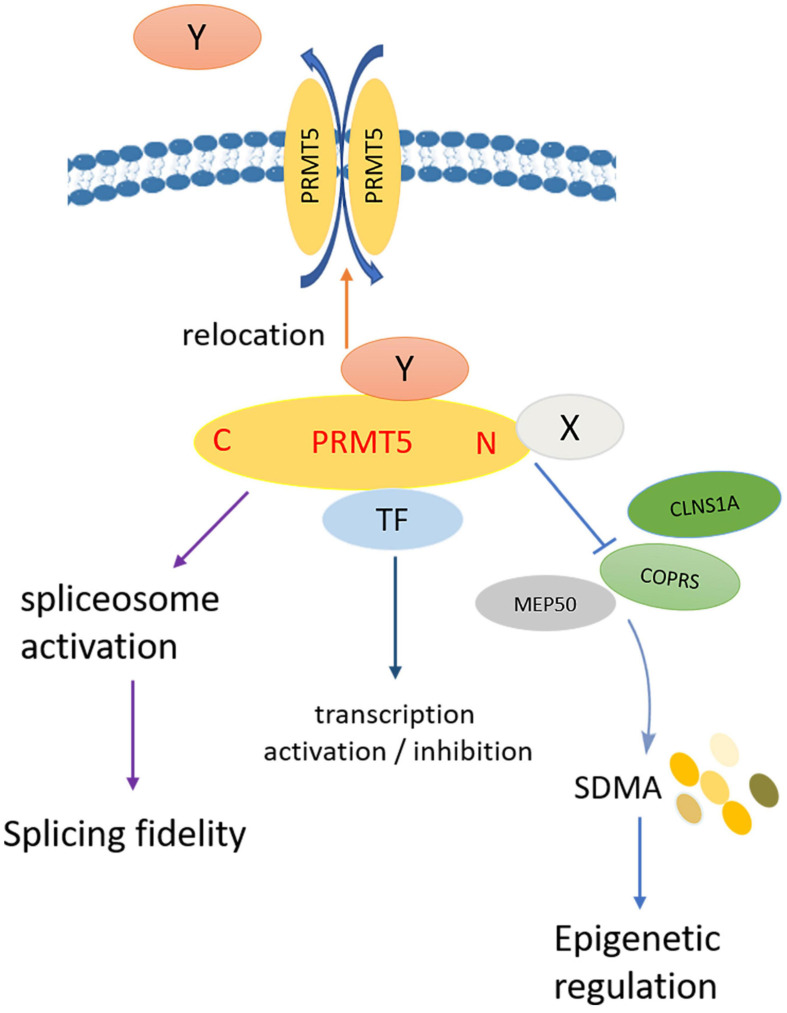
Model diagram of PRMT5 regulating biological functions. Firstly, PIPs directly bind to the N-terminal of PRMT5, which competitively destroys the binding of PRMT5 to COPRS, leading to the change of histone and non-histone arginine residues SDMA level; secondly, PRMT5 binds to transcription factors to play roles in transcription activation or inhibition; thirdly, as a splicing modulator, PRMT5 mediates spliceosome activation, and regulates the fidelity of splicing; fourthly, PIPs affect the subcellular localization of PRMT5, nuclear PRMT5, and cytoplasmic PRMT5 play opposite roles in cell proliferation; at the same time, PRMT5 also affects the subcellular localization of PIPs by methylation of arginine residues.

To sum up, the summarized model diagram of PRMT5 functions is shown in Figure 6. PRMT5 and PIPs participate in the regulation of biological functions through the following aspects: Firstly, PIPs directly bind to the N-terminal of PRMT5, which competitively destroys the binding of PRMT5 to the non-catalytic component of the methylosome complex or the coordinator of PRMT5 and differentiation stimulator (COPRS), leading to the change of histone and non-histone arginine residues SDMA level; secondly, PRMT5 binds to transcription factors to play roles in transcription activation or inhibition; thirdly, as a splicing modulator, PRMT5 mediates spliceosome activation, and regulates the fidelity of splicing; fourthly, PIPs affect the subcellular localization of PRMT5, nuclear PRMT5 and cytoplasmic PRMT5 play opposite roles in cell proliferation. At the same time, PRMT5 also affects the subcellular localization of PIPs by methylation of arginine residues.

### The Application Prospects of PRMT5

Due to the key function of PRMT5 and PIPs in various biological processes, PRMT5 is supposed to act as a therapeutic target. Currently, there is no PRMT5 inhibitor approved for marketing anywhere in the world. Three molecules have entered clinical trials for multiple cancer types^7^, GSK3326595, a substrate competitive inhibitor, currently in the stage of phase II clinical research and JNJ64619178, a SAM (S-adenosyl-l-methionine) mimetic/competitive inhibitor, in phase I clinical stage, and Pfizer’s target drug PF-06939999, also in phase I clinical stage. The rational design of targeted therapy combined with PRMT5 inhibitors has become an effective treatment strategy. Some penitential strategies have been reported as follows.

#### Application of PRMT5 in Liver Diseases

The enzymatic activity of PRMT5 is necessary for the regulation of PPARα and PGC-1α expression and mitochondrial biogenesis. Inhibition of PRMT5 is beneficial to β-oxidation and helps reverse liver steatosis. Targeting PRMT5 may have therapeutic potential for the treatment of fatty liver (Huang L. et al., 2018). Meanwhile, inhibition of PRMT5 significantly reduces the proliferation of HCC cells, but does not affect the proliferation of normal liver cells. PRMT5 inhibition not only promotes apoptosis, but also inhibits HCC cell migration (Fong et al., 2019). DW14800, a PRMT5 inhibitor, can inhibit the self-renewal ability of HCC stem cells and reconstruct hepatocyte specific characteristics in HCC cells. DW14800 has an anti-tumor effect in HCC, for it can increase the expression of HNF4α by reducing the level of H4R3me2s and enhancing the transcription of HNF4α (Zheng et al., 2019). Moreover, GSK3326595 can not only up-regulate the tumor suppressor gene Cdkn1b/p27, but also induce lymphocyte infiltration and MHC II expression, which helps to enhance the anti-tumor immune response. The combination of GSK3326595 and anti-PD-1 immune checkpoint therapy improves the therapeutic effect of HCC (Luo et al., 2021).

#### Application of PRMT5 for T Cell Regulation

PRMT5 inhibitors can inhibit the proliferation, activity and function of CD8 + T cells by up- regulating p53 and weakening Akt pathway, moreover, these inhibitors not only damage tumor cells, but also resist tumor immune response (Strobl et al., 2020). Inhibition of PRMT5 effectively reduces the phosphorylation of STAT1 and the transcription of pro-inflammatory genes, deactivates the cell cycle of T cells and destroys the signal by activating ERK1/2 phosphorylation. Therefore, PRMT5 is considered as a regulator and therapeutic target of T cell response in acute graft-versus-host disease (Snyder et al., 2020). In addition, PRMT5 inhibitor can arrest TCR-induced cell cycle progression at G1/S checkpoint, and reduce IL-2, a cytokine associated with T cell proliferation. PRMT5 inhibitors may contribute to control the expansion of T cells and thus improve the diseases related to T cell activation (Amici et al., 2021).

#### Application of PRMT5 for Multiple Cancers

At present, inhibition of PRMT5 has also become a potential therapy for methionine adenosine phosphorylase (MTAP)-deficient cancers. In MTAP deficient malignant mesothelioma cells, PRMT5 knockdown reduces the expression of E2F1 involved in cell cycle process, and reduces the factors related to epithelial mesenchymal transition. Targeting PRMT5 may represent a promising new treatment strategy for MTAP deleted malignant mesotheliom (Barbarino et al., 2020).

Breast cancer progression, treatment resistance and relapse are thought to originate from breast cancer stem cells (BCSC). PRMT5 is identified as the key modulators for BCSC proliferation and self-renewal. PRMT5 depletion or PRMT5 inhibition will significantly reduce the number of BCSC. The use of small molecule inhibitors of PRMT5 or the downstream targets may be an effective strategy to eliminate this carcinogenic population (Chiang et al., 2017).

In castration-resistant prostate cancer (CRPC), PRMT5 recruits pICln to the androgen receptor (AR) promoter to activate AR transcription, and PRMT5 may be a novel target for CRPC treatment by suppressing expression of AR (Beketova and Fang, 2020).

Arginine methyltransferase inhibitor 1 (AMI-1), a small molecule inhibitor of PRMTs, could enhance the therapeutic effect of cisplatin in lung cancer cells and exhibited protective ability on normal lung epithelial cells. Therefore, the use of AMI-1 to inhibit PRMT5 is a promising therapeutic candidate for improving lung cancer resistance to cisplatin (Bajbouj et al., 2021).

Since non-tumor cells express low levels of PRMT5, specific inhibitors of PRMT5 activity that have been developed may show limited cytotoxicity and high specificity against cancer cells, which may facilitate their use of single drug or combination therapy in new cancer treatment methods that are not responsive to conventional therapy (Bielli et al., 2019).

#### Other Applications of PRMT5

Inhibition of PRMT5 significantly reduces the renal injury and promotes the proliferation of renal tubular cells, at the same time, it reduces the oxidative stress induced by renal ischemia/reperfusion, activates Nrf2/HO-1 signal transduction, and reduces ROS-induced inflammation and apoptosis to prevent ischemia/reperfusion injury (Diao and Chen, 2019). PRMT5 inhibition also attenuates IL-1β-stimulated induced chondrocyte catabolism, and PRMT5 inhibitor EPZ can attenuate cartilage degradation by reducing MAPK and NF-κB signaling (Dong et al., 2020). In addition, the direct dimethylation of cGAS by PRMT5 inhibits cGAS-mediated immune response against DNA viruses, and both EPZ015666 and GSK3326595 can significantly inhibit the replication of HSV-1 virus in infected mice, the inhibition of PRMT5 protects mice from DNA viruses by regulating cGAS/STING signaling pathway. These suggested that PRMT5 inhibitors may be applied to immune system regulation (Ma et al., 2021).

## Conclusion

At present, our knowledge about the mechanism of PRMT5 dysregulation in diseases, especially cancer, is still limited. This study elucidates for the first time the overall functions of PRMT5 together with interacting proteins; the systemic analysis will enrich the biological theory and benefit the development novel PRMT5-targeting therapies.

## Data Availability Statement

The original contributions presented in the study are included in the article/Supplementary Material, further inquiries can be directed to the corresponding author/s.

## Author Contributions

ZL was in charge of the writing of manuscript. CW and HJ were in charge of editing the manuscript. SM was in charge of the revision of manuscript. XL conceived and designed this study, reviewed the manuscript and was responsible for the overall content as guarantor. All authors contributed to the article and approved the submitted version.

## Conflict of Interest

The authors declare that the research was conducted in the absence of any commercial or financial relationships that could be construed as a potential conflict of interest.

## Publisher’s Note

All claims expressed in this article are solely those of the authors and do not necessarily represent those of their affiliated organizations, or those of the publisher, the editors and the reviewers. Any product that may be evaluated in this article, or claim that may be made by its manufacturer, is not guaranteed or endorsed by the publisher.

## Footnotes

1
http://string-db.org/

2
http://www.ebi.ac.uk/intact/

3
http://mint.bio.uniroma2.it/

4
http://www.hprd.org

5
http://omics.bjcancer.org/pina/

6
https://thebiogrid.org/

7
https://clinicaltrials.gov/ct2/results?cond=&term=PRMT5+inhibitor&cntry=&state=&city=&dist=

## Supplementary Material

Supplementary Figure 1 The Upset Plot of the PRMT5 interacting proteins predicted in 6 protein-protein interacting datasets.

Supplementary Table 1 The interacting proteins of PRMT5 predicted in 6 protein-protein interacting databases.

Supplementary Table 2 GO Enrichment analysis of PRMT5 interacting proteins.

Supplementary Table 3 KEGG Enrichment analysis of PRMT5 interacting proteins.

## Abbreviations

PRMTs: Protein arginine methyltransferases
PIPs: PRMT5 interacting proteins
SAM: S-adenosine -L-methionine
SAH: S-adenosine -L-homocysteine
SDMA: symmetric dimethylarginine
PPI: protein-protein interaction
HCC: hepatocellular carcinoma
snRNAs: small nuclear RNAs
snRNPs: small nuclear ribonucleoproteins
SE: exon skipping
ALL: acute lymphoblastic leukemia
BCR: B-cell receptor
GAK: Cyclin G-related kinase
CAPG: macrophage-capping protein
EMT: Epithelial mesenchymal transition
ROS: reactive oxygen species
MTAP: methionine adenosine phosphorylase.
