# Supplementary figures and images for: Protein Arginine Methyltransferase 5 Functions via Interacting Proteins
Source: Front Cell Dev Biol. 2021 Aug 27;9:725301. doi: 10.3389/fcell.2021.725301 (PMC8432624; doi:10.3389/fcell.2021.725301)

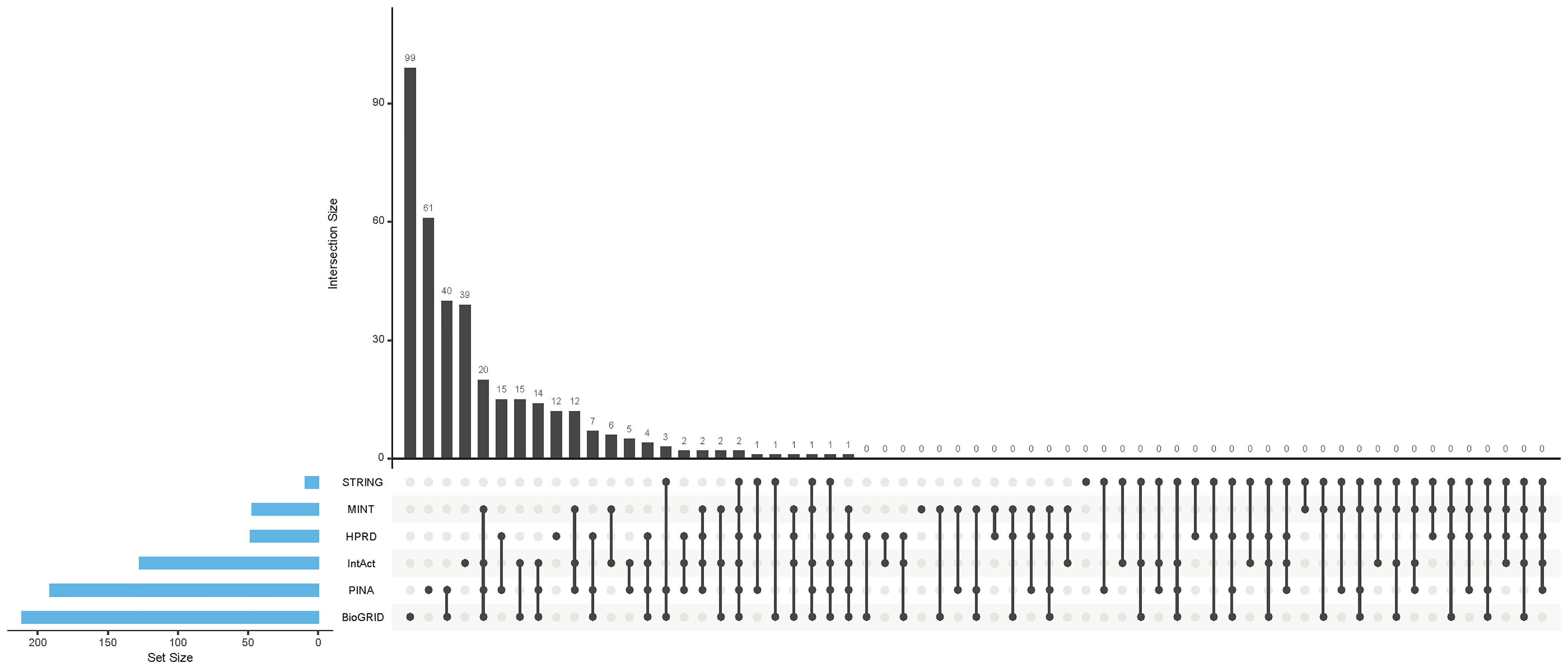

Supplement: Supplementary Figure 1 — The Upset Plot of the PRMT5 interacting proteins predicted in 6 protein-protein interacting datasets. [file Image_1.TIFF]
