# Supplementary material for: Protein Arginine Methyltransferase 5 Functions via Interacting Proteins
Source: Front Cell Dev Biol. 2021 Aug 27;9:725301. doi: 10.3389/fcell.2021.725301 (PMC8432624; doi:10.3389/fcell.2021.725301)
Supplement: Supplementary Table 1 — The interacting proteins of PRMT5 predicted in 6 protein-protein interacting databases. [file Table_1.DOCX]

Supplementary Table 1. The interacting proteins of PRMT5 predicted in each database

| **STRING** | **IntAct** | **MINT** | **HPRD** | **PINA** | **BioGRID** |
| --- | --- | --- | --- | --- | --- |
| WDR77 | WDR77 | AGO1 | YWHAQ | AJUBA | ACO2 |
| CLNS1A | CLNS1A | AGO2 | CLNS1A | ARGLU1 | ACTN4 |
| PRMT1 | H4-16 | CALU | OLA1 | ARID1B | AGO3 |
| COPRS | PRMT5 | CBX5 | JAK1 | ARRB1 | AGO4 |
| SNRPD1 | YWHAZ | CDYL2 | JAK2 | ARRB2 | AGR2 |
| HIST4H4 | E2F1 | COPRS | JAK3 | C1QBP | AHSA1 |
| CDK4 | RBM23 | E2F1 | WDR77 | C1orf63 | AJUBA |
| HIST1H4A | SRGAP2 | EZH2 | MBP | CALU | ALKBH3 |
| HIST2H2AC | DNMT3A | FAM76B | SNRPD1 | CAPN1 | APEX1 |
|  | CTDP1 | GRHL3 | SNRPD3 | CBX1 | ARGLU1 |
|  | EPHB6 | HOXC4 | SNRPB | CBX5 | ARID1B |
|  | GAS8 | JUN | SUPT5H | CCND1 | ARRB1 |
|  | POLR2A | LENG8 | TYK2 | CDK4 | ARRB2 |
|  | EIF4A3 | MCRS1 | PRMT5 | CDYL2 | ATG16L1 |
|  | CACNB2 | NELFCD | CAPN1 | CLK1 | BCL2L14 |
|  | EHHADH | PIAS4 | CTDP1 | CLK3 | BIRC3 |
|  | ELOA | RBM23 | SSTR1 | CLNS1A | BRCA1 |
|  | CDC37 | CTDP1 | YWHAG | COBRA1 | BRD4 |
|  | KANK2 | YWHAZ | PHYHIPL | COPRS | C14orf178 |
|  | LNX1 | GTPBP2 | FBL | COPS4 | CALU |
|  | ZMYND19 | TRIB3 | TUBB | COPS7A | CAPN1 |
|  | WDYHV1 | NUDCD2 | C1QBP | CSNK1E | CARM1 |
|  | C1orf109 | ARGLU1 | TUBA | CTDP1 | CCAR2 |
|  | VCAM1 | LUC7L | PRMT1 | CTNNBL1 | CCDC33 |
|  | ESR2 | LDHAL6B | SNRPN | CUL3 | CCND1 |
|  | HOXA9 | RSRP1 | SMN1 | CUL4A | CDK19 |
|  | NFE4 | SPAG8 | DDX20 | DCAF8 | CDK4 |
|  | COPRS | ING5 | GEMIN4 | DDX20 | CDK8 |
|  | CDYL2 | CCAR2 | MACROH2A1 | DDX21 | CDYL2 |
|  | GRHL3 | H3-5 | PHF14 | DEK | CFLAR |
|  | NUDCD2 | SF3A3 | ADPRT | DET1 | CHTOP |
|  | TRIB3 | CLNS1A | TOP1 | DHX15 | CIRBP |
|  | GTPBP2 | CLK3 | DDX21 | DIO3 | CLK1 |
|  | CRY1 | CLK1 | Ku antigen | DNMT3A | CLK3 |
|  | RIOK1 | YWHAB | HSP70B | DST | CLNS1A |
|  | SKI | YWHAQ | HDAC1 | E2F1 | COPRS |
|  | SMN1 | MAGEB2 | HP1BP3 | EIF2C1 | COPS4 |
|  | IVL | PRMT5 | RCC1 | EIF2C2 | COPS7A |
|  | PLEC | PRPF38A | DEK | EIF2C3 | CRY1 |
|  | JAK2 | SCN5A | H1C | EIF2C4 | CSNK1E |
|  | SNRPB | SLU7 | H1A | EIF4A3 | CTDP1 |
|  | YWHAQ | SNAI1 | RAN | EPB41L3 | CTNNBL1 |
|  | YWHAB | SNRNP70 | CBX5 | ERCC3 | CTPS1 |
|  | SNRPD1 | WDR77 | CBX1 | EZH2 | CUL3 |
|  | SCN5A | ZNF2 | H3-3B | FAM76B | CUL4A |
|  | PIAS4 | ZNF436 | H2be | FBL | CUL4B |
|  | MAGEB2 | ZUP1 | H2AX | FBXO25 | CUL7 |
|  | CALU |  | H4C5 | FBXO6 | CYLD |
|  | SLU7 |  |  | FN1 | DCAF4L2 |
|  | SNRNP70 |  |  | FOXK1 | DCAF8 |
|  | HOXC4 |  |  | GEMIN2 | DDB1 |
|  | CLK1 |  |  | GEMIN4 | DIO3 |
|  | CLK3 |  |  | GRHL3 | DNMT1 |
|  | SF3A3 |  |  | GTF2B | DNMT3A |
|  | EZH2 |  |  | GTF2F1 | DST |
|  | FAM76B |  |  | GTF2F2 | DUSP14 |
|  | H3-5 |  |  | GTF2H1 | DYNC1H1 |
|  | ZDHHC17 |  |  | GTPBP2 | DYNLT1 |
|  | NELFCD |  |  | H2AFX | DYRK1A |
|  | CCAR2 |  |  | H2AFY | EFTUD2 |
|  | PRPF38A |  |  | H3F3A | EGLN3 |
|  | ING5 |  |  | H3F3C | EIF2S1 |
|  | PHYHIP |  |  | HDAC1 | EIF4A3 |
|  | ZUP1 |  |  | HDAC5 | EP300 |
|  | MCRS1 |  |  | HDGF | EPB41L3 |
|  | LENG8 |  |  | HECW2 | ESR2 |
|  | SPAG8 |  |  | HIST1H1A | EWSR1 |
|  | ZNF2 |  |  | HIST1H1C | EZH1 |
|  | RSRP1 |  |  | HIST1H2AB | EZH2 |
|  | TRIM54 |  |  | HIST1H2BC | FAM76B |
|  | LDHAL6B |  |  | HIST1H3A | FARP2 |
|  | ZNF436 |  |  | HIST1H4A | FBL |
|  | LUC7L |  |  | HIST2H2AC | FBXO25 |
|  | OLA1 |  |  | HOXA9 | FBXO6 |
|  | ARGLU1 |  |  | HOXC4 | FN1 |
|  | SMAD7 |  |  | HP1BP3 | FOXK1 |
|  | CUL4A |  |  | HSP90AA1 | FUS |
|  | CUL4B |  |  | HSPA6 | G3BP1 |
|  | TNFRSF10A |  |  | Hnrnph1 | GAN |
|  | PLEKHA7 |  |  | ING5 | GAR1 |
|  | HDAC3 |  |  | INTS1 | GHET1 |
|  | RBFOX2 |  |  | INTS10 | GLI1 |
|  | FARP2 |  |  | INTS12 | GLIS3 |
|  | SNAI1 |  |  | INTS2 | GRHL3 |
|  | FN1 |  |  | INTS6 | GTPBP2 |
|  | ESR1 |  |  | JAK1 | H3F3C |
|  | JUN |  |  | JAK2 | h4-A |
|  | POLR2C |  |  | JAK3 | HDAC1 |
|  | RAB5A |  |  | KHDRBS2 | HDAC5 |
|  | FBL |  |  | KIAA1967 | HDGF |
|  | SFPQ |  |  | LDHAL6B | HECW2 |
|  | GATA3 |  |  | LENG8 | HEXIM1 |
|  | ELK4 |  |  | LSM11 | HHV8GK18_gp81 |
|  | ARRB2 |  |  | LUC7L | HIST1H3A |
|  | CBX5 |  |  | MAGEB2 | HIST1H3E |
|  | ARRB1 |  |  | MAGOH | HIST1H4A |
|  | CSNK1E |  |  | MBD2 | HIST2H2AC |
|  | RPL15 |  |  | MBP | HIST3H3 |
|  | SNRPE |  |  | MCRS1 | HIST4H4 |
|  | LSM6 |  |  | MED17 | HNRNPH1 |
|  | GRB2 |  |  | MED27 | HOXA9 |
|  | SNRPN |  |  | MEF2D | HOXC4 |
|  | SMAD3 |  |  | MYOD1 | HRAS |
|  | PRR5 |  |  | MYOG | HSP90AA1 |
|  | RBM3 |  |  | MYSM1 | HSPA8 |
|  | IFT88 |  |  | NCL | ID2 |
|  | MTA1 |  |  | NFE4 | ILF3 |
|  | SMAD4 |  |  | NUDCD2 | ING2 |
|  | CUL3 |  |  | OLA1 | ING5 |
|  | CIRBP |  |  | PAN2 | JAK1 |
|  | SMAD2 |  |  | PARP1 | JAK2 |
|  | LRRK2 |  |  | PAXIP1 | JAK3 |
|  | SETX |  |  | PHF14 | KDM1A |
|  | METTL3 |  |  | PHYHIP | KDM2B |
|  | CCDC33 |  |  | PIAS4 | KDM3A |
|  | MAP3K14 |  |  | PIK3C3 | KDM3B |
|  | BCL2L14 |  |  | POLR2A | KDM4A |
|  | TINAGL1 |  |  | POLR2B | KDM4C |
|  | GABARAPL1 |  |  | POLR2C | KDM6B |
|  | AGO3 |  |  | POLR2D | KHDRBS2 |
|  | AGO4 |  |  | POLR2E | KIAA1429 |
|  | RBFOX1 |  |  | POLR2H | KRAS |
|  | GAR1 |  |  | POLR2J | LARP7 |
|  | MYEF2 |  |  | PRDM1 | LDHAL6B |
|  | AGO2 |  |  | PRMT1 | LENG8 |
|  | AGO1 |  |  | PRPF38A | LRWD1 |
|  | EPB41L3 |  |  | PRR5 | LSM11 |
|  |  |  |  | RAN | LSM4 |
|  |  |  |  | RBM23 | LSM6 |
|  |  |  |  | RCC1 | LTN1 |
|  |  |  |  | RDBP | LUC7L |
|  |  |  |  | RECQL5 | MAGEB2 |
|  |  |  |  | RELA | MAGOH |
|  |  |  |  | SCN5A | MAP3K14 |
|  |  |  |  | SF3A3 | MBD1 |
|  |  |  |  | SHC1 | MBD2 |
|  |  |  |  | SIN3A | MBP |
|  |  |  |  | SIRT7 | MCM5 |
|  |  |  |  | SKI | MCRS1 |
|  |  |  |  | SLU7 | MEF2D |
|  |  |  |  | SMARCA2 | METTL3 |
|  |  |  |  | SMARCA4 | Mib1 |
|  |  |  |  | SMARCB1 | MRPL11 |
|  |  |  |  | SMARCC1 | MTF1 |
|  |  |  |  | SMARCE1 | MYEF2 |
|  |  |  |  | SMN1 | MYOD1 |
|  |  |  |  | SMURF1 | MYOG |
|  |  |  |  | SNAI1 | MYSM1 |
|  |  |  |  | SNRNP70 | NBN |
|  |  |  |  | SNRPB | NCBP1 |
|  |  |  |  | SNRPD1 | NCL |
|  |  |  |  | SNRPD3 | NELFCD |
|  |  |  |  | SNRPN | NFE4 |
|  |  |  |  | SPAG8 | NR2C2 |
|  |  |  |  | SRGAP2 | NRAS |
|  |  |  |  | SSTR1 | NSFL1C |
|  |  |  |  | SUPT5H | NTRK1 |
|  |  |  |  | SUPT6H | NUDCD2 |
|  |  |  |  | TCEA1 | OLA1 |
|  |  |  |  | TCF3 | Osgep |
|  |  |  |  | TERF1 | PAN2 |
|  |  |  |  | TERF2 | PAXIP1 |
|  |  |  |  | TH1L | PDCD4 |
|  |  |  |  | TLE4 | PDGFRA |
|  |  |  |  | TOP1 | PDIA6 |
|  |  |  |  | TRAF4 | PHB |
|  |  |  |  | TRIB3 | PHF1 |
|  |  |  |  | TUBA1A | PHF2 |
|  |  |  |  | TUBB | PHF8 |
|  |  |  |  | TYK2 | PHYHIP |
|  |  |  |  | UBC | PIH1D1 |
|  |  |  |  | UBD | PIK3C3 |
|  |  |  |  | UBE2Q2 | PLEKHA4 |
|  |  |  |  | UBL4A | POLR2A |
|  |  |  |  | VASP | POLR2C |
|  |  |  |  | VCAM1 | PPCS |
|  |  |  |  | VCP | PPIE |
|  |  |  |  | WDR77 | PRDM1 |
|  |  |  |  | WHSC2 | PRMT5 |
|  |  |  |  | WWOX | RANBP2 |
|  |  |  |  | XRCC5 | RBM23 |
|  |  |  |  | XRCC6 | RELA |
|  |  |  |  | YWHAB | RIOK1 |
|  |  |  |  | YWHAG | RPL23 |
|  |  |  |  | YWHAQ | RRP12 |
|  |  |  |  | YWHAZ | SBNO1 |
|  |  |  |  | ZDHHC17 | SF3A3 |
|  |  |  |  | ZNF2 | SHARPIN |
|  |  |  |  | ZNF224 | SIN3A |
|  |  |  |  | ZNF436 | SMARCA2 |
|  |  |  |  | ZUFSP | SMARCA4 |
|  |  |  |  |  | SMARCB1 |
|  |  |  |  |  | SMARCE1 |
|  |  |  |  |  | SNRNP70 |
|  |  |  |  |  | SNRPB |
|  |  |  |  |  | SNRPD1 |
|  |  |  |  |  | SREBF1 |
|  |  |  |  |  | ST7 |
|  |  |  |  |  | STUB1 |
|  |  |  |  |  | SUPT5H |
|  |  |  |  |  | TRAF4 |
|  |  |  |  |  | TRIM21 |
|  |  |  |  |  | TYK2 |
|  |  |  |  |  | UBA2 |
|  |  |  |  |  | UHRF1 |
|  |  |  |  |  | VASP |
|  |  |  |  |  | WDR5 |
|  |  |  |  |  | WDR77 |
|  |  |  |  |  | ZNF224 |
|  |  |  |  |  | ZNF326 |
|  |  |  |  |  | ZNHIT6 |
